# Hydrogen spillover in complex oxide multifunctional sites improves acidic hydrogen evolution electrocatalysis

**DOI:** 10.1038/s41467-022-28843-2

**Published:** 2022-03-04

**Authors:** Jie Dai, Yinlong Zhu, Yu Chen, Xue Wen, Mingce Long, Xinhao Wu, Zhiwei Hu, Daqin Guan, Xixi Wang, Chuan Zhou, Qian Lin, Yifei Sun, Shih-Chang Weng, Huanting Wang, Wei Zhou, Zongping Shao

**Affiliations:** 1 State Key Laboratory of Materials-Oriented Chemical Engineering, College of Chemical Engineering, Nanjing Tech University, Nanjing, 211800 China; 2 Department of Chemical Engineering, Monash University, Clayton, VIC 3800 Australia; 3 Monash Centre for Electron Microscopy, Monash University, Clayton, VIC 3800 Australia; 4 School of Environmental Science and Engineering, Key Laboratory for Thin Film and Microfabrication of the Ministry of Education, Shanghai Jiao Tong University, Shanghai, 200240 China; 5 Max Planck Institute for Chemical Physics of Solids, Nothnitzer Strasse 40, 01187 Dresden, Germany; 6 College of Energy, Xiamen University, Xiamen, 361102 China; 7 National Synchrotron Radiation Research Center, 101 Hsin-Ann Road, Hsinchu, 30076 Taiwan; 8 WA School of Mines: Minerals, Energy and Chemical Engineering, Curtin University, Perth, WA 6845 Australia

**Keywords:** Electrocatalysis, Hydrogen energy, Electrocatalysis

## Abstract

Improving the catalytic efficiency of platinum for the hydrogen evolution reaction is valuable for water splitting technologies. Hydrogen spillover has emerged as a new strategy in designing binary-component Pt/support electrocatalysts. However, such binary catalysts often suffer from a long reaction pathway, undesirable interfacial barrier, and complicated synthetic processes. Here we report a single-phase complex oxide La_2_Sr_2_PtO_7+δ_ as a high-performance hydrogen evolution electrocatalyst in acidic media utilizing an atomic-scale hydrogen spillover effect between multifunctional catalytic sites. With insights from comprehensive experiments and theoretical calculations, the overall hydrogen evolution pathway proceeds along three steps: fast proton adsorption on O site, facile hydrogen migration from O site to Pt site via thermoneutral La-Pt bridge site serving as the mediator, and favorable H_2_ desorption on Pt site. Benefiting from this catalytic process, the resulting La_2_Sr_2_PtO_7+δ_ exhibits a low overpotential of 13 mV at 10 mA cm^−2^, a small Tafel slope of 22 mV dec^−1^, an enhanced intrinsic activity, and a greater durability than commercial Pt black catalyst.

## Abstract (web summary)

While renewable H_2_ production offers a promising route for clean energy production, there is an urgent need to improve catalyst performances. Here, authors design a Pt-containing complex oxide that utilizes atomic-scale hydrogen spillover to promote H_2_ evolution electrocatalysis in acidic media.

## Introduction

Accelerated exhaustion of fossil fuels accompanied with ever-increasing environmental issues has raised great concerns about the exploitation of renewable energy sources (e.g., solar and wind power)^1,2^. To overcome the intermittent nature of the renewable energy, an attractive prospect is to store them in the form of chemical bonds in certain molecular fuels^3,4^. Among various chemical fuels, hydrogen (H_2_) has been pursued as the future sustainable energy alternatives to fossil fuels in view of high gravimetric energy density and carbon-free characteristics^5–7^. Therefore, finding a way to produce hydrogen efficiently is crucial for the future hydrogen economy. Electrochemical water splitting powered by renewable energy offers a cost-effective and promising approach for clean hydrogen production with high purity^8–10^. Water splitting in acidic solid polymer electrolytes is more efficient than alkaline electrolysis because it holds some notable superiority such as greater energy efficiency, higher current density, lower crossover of gases and more compact system design^11,12^. As the cathodic reaction in water splitting, the hydrogen evolution reaction (HER) is sluggish and requires an efficient electrocatalyst to expedite the rate. Currently, although a host of non-platinum candidate materials for HER were studied, the metallic platinum (Pt) is still considered as “the Holy Grail” of HER electrocatalyst in acidic media with a nearly-zero onset overpotential and fast kinetics owing to its favorable hydrogen binding energy^13,14^. However, the natural scarcity, high cost and poor durability limit its large-scale commercial applications of water electrolyzers^15,16^. As a consequence, it is necessary to improve the intrinsically catalytic ability and the utilization efficiency of Pt for HER.

**Fig. 1:**
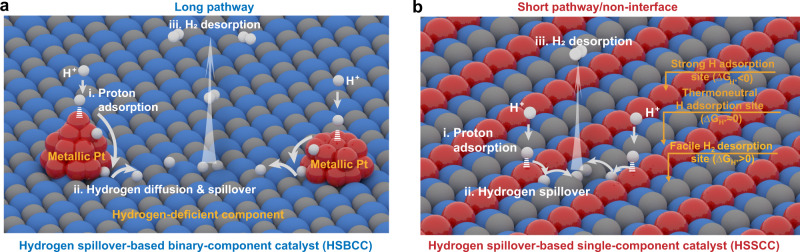
Schematic illustration of the hydrogen spillover on two-type catalyst systems for HER in acidic media. **a** The conventional hydrogen spillover-based binary-component catalyst (HSBCC) system by coupling hydrogen-enriched Pt-based nanocrystals with hydrogen-deficient component. Red balls represent Pt atoms and blue and gray balls represent compounds. **b** Hydrogen spillover-based single-component catalyst (HSSCC) system with atomic-level multiple catalytic sites. Red, blue and gray balls represent strong H adsorption, thermoneutral H adsorption and facile H_2_ desorption sites, respectively.

Hitherto, numerous efforts have been made to design Pt-based HER catalysts in acid^17–24^. For example, the catalytic efficiency of Pt metal could be boosted by the size^17^, composition^18^, morphology^19^, and crystal phase-engineering strategies^20^. Besides, hybridizing Pt with another component is also an effective way to achieve high-performance HER electrocatalysts via hydrogen spillover phenomenon^21–24^. Recently, hydrogen spillover opens new opportunities for improving the HER activity of binary metal/support catalysts with hydrogen-enriched Pt nanocrystals and hydrogen-deficient components, such as WO_3-x_^21^, SiO_2_^22^, RuCeO_x_^23^, CoP^24^. As schematically illustrated in Fig. 1a, hydrogen spillover-based binary-component catalyst (HSBCC) involves three main steps: (1) the strong proton (H^+^) adsorption on metals (e.g., Pt with Δ*G*_H-metal_ < 0), (2) the interfacial H diffusion and spillover from metals to supports, and (3) efficient H_2_ desorption on supports (Δ*G*_H-support_ > 0)^24^. Nonetheless, considerable barriers need to be overcome for the hydrogen spillover process in HSBCC systems due to long reaction path and undesirable interfacial resistance within two components (e.g., Schottky barrier and unmatched lattice space)^2,25–27^. Furthermore, HSBCCs always suffer from complicated synthesis processes, which are disadvantageous for cost-effective and large-scale fabrication. In light of the short reaction path and interface-free feature in single-component catalysts^2,25,28,29^, the creation of atomic-level multiple catalytic sites for strong H^+^ adsorption, thermo-neutral H adsorption and facile H_2_ desorption simultaneously in hydrogen spillover-based single-component catalyst (HSSCC) system (as illustrated in Fig. 1b), is highly desirable to boost the acidic HER activity; yet still, such single-phase catalysts have not been reported so far.

Multi-metal oxides have attracted great interest in many catalytic applications by virtue of their structural and compositional flexibility^30–32^. The multiple elements and variable crystal structures could endow multi-metal oxides with some unique geometrical and electronic properties, consequently tailoring the binding behavior of reaction intermediates and promoting their electrocatalytic activities^30–32^. Inspired by aforementioned considerations, here we demonstrate a unique family of complex metal oxide prepared by a facile solid-phase reaction method, La_2_Sr_2_PtO_7+δ_, as a highly active and durable HER electrocatalyst in acid media. This complex oxide crystallizes in a hexagonal structure with alternating layers of [La_2_PtO_6_] containing isolated Pt^IV^O_6_ octahedra and [Sr_2_O_1+δ_] slabs. The La_2_Sr_2_PtO_7+δ_ oxide displays a remarkable HER activity with a low overpotential of 13 mV at 10 mA cm^−2^ and a small Tafel slope of 22 mV dec^−1^ in 0.5 M H_2_SO_4_, superior to state-of-the-art HSBCCs and other Pt-based catalysts ever reported. In addition, the La_2_Sr_2_PtO_7+δ_ shows significant enhancement in the intrinsic activity and operational durability as compared with the commercial Pt black catalyst. Comprehensive experiments and electrochemical measurements were performed to support the possible occurrence of hydrogen spillover in La_2_Sr_2_PtO_7+δ_. First-principles calculations suggest that the hydrogen adsorption at La-Pt bridge site in La_2_Sr_2_PtO_7+δ_ is nearly thermo-neutral, which could serve as the mediators for favorable hydrogen spillover and accordingly result in exceptionally high activity. Specifically, a unique synergistic mechanism of multi-function catalytic sites in La_2_Sr_2_PtO_7+δ_ involving hydrogen spillover for HER was proposed: the O site serves as the proton enrichment, the thermo-neutral La-Pt bridge site serves as the mediator for favorable hydrogen spillover/migration from O site to Pt site, and Pt site favors the final H_2_ desorption. This work opens a new avenue for the design of high-performance HER catalysts in acid media though hydrogen spillover among multi-function-site synergy in single component.

## Results

### Crystal structure and morphology

**Fig. 2:**
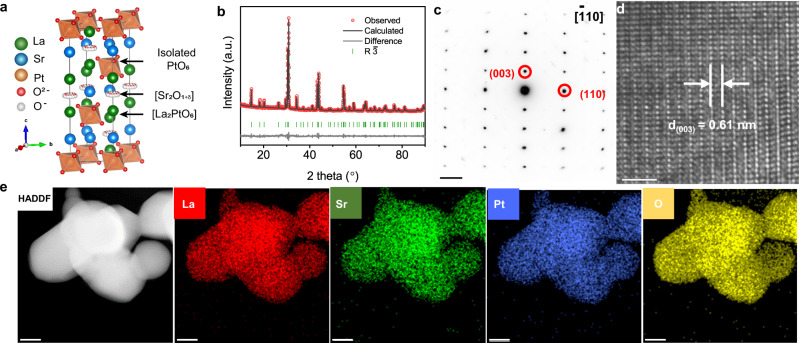
Crystal structure and morphology characterization. **a** Refined XRD profile of La_2_Sr_2_PtO_7+δ_. **b** Schematic presentation of La_2_Sr_2_PtO_7+δ_ structure. **c** SAED pattern along the [−110] direction and **d** the corresponding HRTEM image of La_2_Sr_2_PtO_7+δ_. **e** HAADF-STEM and the corresponding elemental mapping images of La_2_Sr_2_PtO_7+δ_. Scale bar in **c** is 2 nm^−1^, in **d** is 2 nm and in **e** is 100 nm.

The kind of complex oxide La_2_Sr_2_PtO_7+δ_ crystallizes in a hexagonal structure containing oxidized Pt ion as the B-site cation. Actually, La_2_Sr_2_PtO_7+δ_ is the *n* = 2 member of the general family of [A’_2_O_1+δ_][A_n_B_n-1_O_3n_] hexagonal perovskites with *n* representing the number of AO_3_ successive layers^33^. Along the *c*-axis, the structure of La_2_Sr_2_PtO_7+δ_ can be presented by the uniform stacking of two LaO_3_ layers and one Sr_2_O_1+δ_ layer (Fig. 2a). In the [La_2_PtO_6_] slab, the Pt ions occupy the octahedral sites between the neighboring LaO_3_ layers to form isolated PtO_6_ units. The La_2_Sr_2_PtO_7+δ_ oxide powder were successfully prepared by conventional solid-state synthesis, and the crystal structure was initially verified by X-ray diffraction (XRD). Rietveld refinement of the XRD pattern reveals that the La_2_Sr_2_PtO_7+δ_ adopts a hexagonal structure with a space group of R-3, and lattice parameters of *a* = *b* = 5.7913(2) Å, *c* = 18.1097(7) Å (Fig. 2b and Supplementary Table 1), which well agrees with previous study^33^. The phase structure was further confirmed by the selected area electron diffraction (SAED) pattern along the [−110] direction and the corresponding high-resolution transmission electron microscopy (HRTEM) image. The SAED pattern in Fig. 2c reflects the hexagonally arranged diffraction spots of [−110] zone axis. A lattice fringe with lattice spacing of 0.61 nm was seen in the HRTEM image (Fig. 2d), corresponding to the (003) plane of La_2_Sr_2_PtO_7+δ_ oxide. Besides, the morphology of La_2_Sr_2_PtO_7+δ_ powder was examined by scanning electron microscopy (SEM). Some chunks composed of micrometer-sized particles were observed (Supplementary Fig. 1), suggesting the bulk nature of the as-synthesized La_2_Sr_2_PtO_7+δ_ oxide by solid-state reaction method. As shown in Fig. 2e, the high-angle annular dark-field scanning transmission electron microscopy and elemental mapping images demonstrate the homogeneous distribution of all elements in the as-prepared La_2_Sr_2_PtO_7+δ_ material. For a direct comparison in this work, commercial Pt black catalyst was also included and the pure phase structure was confirmed by XRD patterns (Supplementary Fig. 2). The Pt black catalyst has broad XRD peaks, implying nanocrystalline feature of the metallic Pt as evidenced by small nanoparticles (~5 nm) in TEM image (Supplementary Fig. 3).

### Electronic structure

**Fig. 3:**
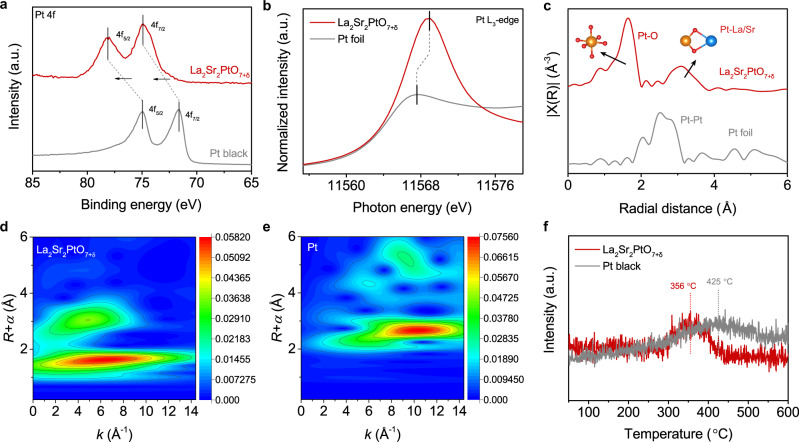
Electronic structure characterization. **a** The high-resolution Pt 4*f* XPS spectra of La_2_Sr_2_PtO_7+δ_ and Pt black. **b** Pt *L*_3_-edge XANES spectra and **c** *K*^3^-weighted Fourier transform EXAFS spectra of the La_2_Sr_2_PtO_7+δ_ and Pt foil. Wavelet transform for the *K*^3^-weighted EXAFS spectra of **d** La_2_Sr_2_PtO_7+δ_ and **e** Pt foil. **f** H_2_-TPD profiles of La_2_Sr_2_PtO_7+δ_ and Pt black.

X-ray photoelectron spectroscopy (XPS) and X-ray absorption spectroscopy (XAS) were carried out to explore the surface chemical state and electronic structure of La_2_Sr_2_PtO_7+δ_. Supplementary Fig. 4 presents the full XPS spectrum of La_2_Sr_2_PtO_7+δ_, which demonstrates the existence of La, Sr, Pt, and O elements on the surface. As seen from the high-resolution Pt *4f* core level spectra in Fig. 3a, two peaks at 74.9 and 78.1 eV were observed, which could be ascribed to the Pt *4f*_*7/2*_ and Pt *4f*_*5/2*_ orbitals of oxidized Pt (IV) species^13,23,34^. Notably, in contrast to the Pt *4f* spectra of commercial Pt black, no signals from metallic Pt at 71.4 and 74.7 eV were detected, indicating the absence of metallic Pt in the La_2_Sr_2_PtO_7+δ_.To further confirm the oxidation state of Pt ions in La_2_Sr_2_PtO_7+δ_, X-ray absorption near-edge structure (XANES) spectra were also collected along with standard Pt foil as a reference (Fig. 3b). XANES spectrum at the *5d* *L*_3_-edge is highly sensitive to the valence state of *5d* elements: an increase of the valence state of the *5d* metal ion by one causes a shift of the *L*_3_ spectra by more than one eV toward higher energies^35,36^. The intensity of white line peak in Pt *L*_3_-edge XANES spectra associates with the electronic transition from *2p*_*3/2*_ to unoccupied *5d* states and discloses the oxidation state of Pt species^16,17^. The white-line intensity of the La_2_Sr_2_PtO_7+δ_ is much higher than that of the metallic Pt foil, suggesting Pt oxide species in La_2_Sr_2_PtO_7+δ_^16,17^. Moreover, the white line of Pt-*L*_3_ of La_2_Sr_2_PtO_7+δ_ locates at about 1.5 eV higher in energy than that of Pt foil indicating further higher oxidation state of the former. It is also well known that the extended X-ray absorption fine structure (EXAFS) is sensitive tool to uncover the local coordination of 5d elements^37,38^. Figure 3c shows the *k*^*3*^-weighted Fourier transform (FT) curves at R space of Pt *L*_3_-edge EXAFS spectra for La_2_Sr_2_PtO_7+δ_ in comparison with the Pt foil reference. The most intense peak at 1.64 Å for La_2_Sr_2_PtO_7+δ_ was detected, corresponding to Pt-O bond^17,39^. Also, the isolated PtO_6_ octahedra in La_2_Sr_2_PtO_7+δ_ is evidenced by the absence of the Pt–Pt coordination at ~2.52 Å in the first-shell region relative to Pt foil^17,40^. To visually explore the coordination conditions of Pt, a more powerful wavelet transform (WT) analysis was performed to directly reflect the structure information in the resolution of R space and k space. As shown in Fig. 3d, The WT intensity maximum of La_2_Sr_2_PtO_7+δ_ occurs near R space of 1.7 Å and k space of 6.5 Å^−1^, confirming the coordination structure of Pt-O bonds in the first coordination shell. As for Pt foil, a new WT intensity maximum near 2.7 Å and 10.4 Å^−1^ appears in Fig. 3e, which is associated with Pt-Pt bonding. Based on previous studies, the *5d* orbital of the Pt sites in a highly oxidized state can hybridize with the H *1s* orbital to form weak Pt-H bonds, giving rise to enhanced intrinsic activity and facile H_2_ evolution^13,41^. Accordingly, the unique electronic structure of oxidized Pt sites in La_2_Sr_2_PtO_7+δ_ is expected to help tailor the hydrogen binding energy on the catalyst surface and thereby improve the catalytic activity. Hydrogen temperature-programmed desorption (H_2_-TPD) measurements were carried out to investigate the Pt-H binding capability of La_2_Sr_2_PtO_7+δ_ and Pt black^42,43^. In Fig. 3f, the H_2_ desorption process of Pt black occurs within the temperature window of 276–558 °C with a peak at 425 °C. The La_2_Sr_2_PtO_7+δ_ shows lower desorption peak temperature of 356 °C, indicative of the weakened hydrogen binding energy and easier H_2_ desorption. Combining above analysis, the Pt in La_2_Sr_2_PtO_7+δ_ is in an oxidized state (~Pt^4+^), which is beneficial for H_2_ desorption.

### Electrocatalytic HER performance in acid

**Fig. 4:**
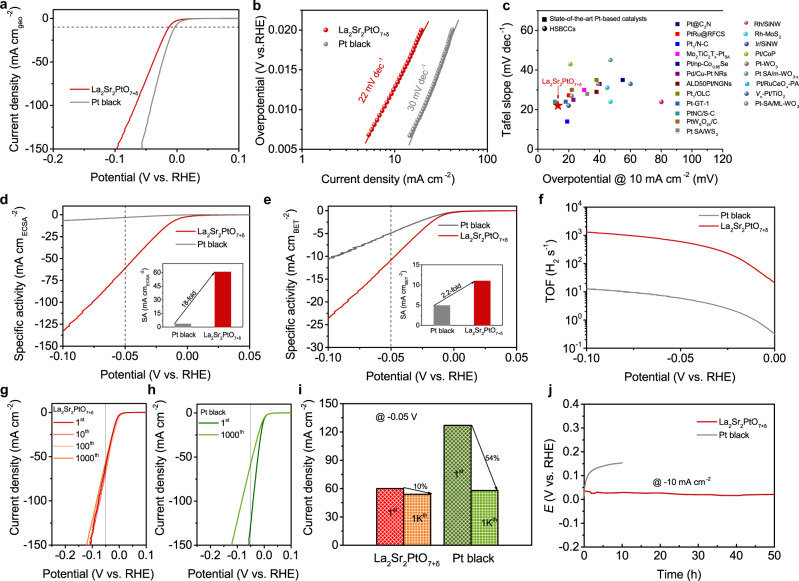
Electrocatalytic HER performance in acid. **a** Polarization curves of La_2_Sr_2_PtO_7+δ_ and Pt black in an Ar-saturated 0.5 M H_2_SO_4_ solution with a scan rate of 5 mV s^−1^. **b** Tafel plots of La_2_Sr_2_PtO_7+δ_ and Pt black. **c** HER activity comparison between La_2_Sr_2_PtO_7+δ_ and HSBCCs and other state-of-the-art Pt-based catalysts ever reported. Specific activity normalized to **d** ECSA and **e** RSA of La_2_Sr_2_PtO_7+δ_ and Pt black as a function of applied potential. Inset: specific activity at the overpotential of *η* = 0.05 V. **f** The relationship between TOF and the tested potentials of La_2_Sr_2_PtO_7+δ_ and Pt black in 0.5 M H_2_SO_4_ solution. **g** Polarization curves of La_2_Sr_2_PtO_7+δ_ initially, as well as after 10, 100, and 1000 cycles. **h** Polarization curves of Pt black initially and after 1000 cycles. **i** Current density comparison at −0.05 V vs. RHE initially and after 1000 cycles for La_2_Sr_2_PtO_7+δ_ and Pt black. **j** Chronopotentiometry response of La_2_Sr_2_PtO_7+δ_ and Pt black at a constant cathodic current density of 10 mA cm^−2^.

To evaluate the acidic HER electrocatalytic performance of La_2_Sr_2_PtO_7+δ_, we conducted electrochemical measurements in 0.5 M H_2_SO_4_ solution using a standard three-electrode configuration. The commercial Pt black was also tested under identical conditions for comparison. If not specified otherwise, all potentials in this work were *iR*-corrected to remove the ohmic drop across the electrolyte and referenced to a reversible hydrogen electrode (RHE, see Supplementary Fig. 5 for calibration). As seen from the polarization curves in Fig. 4a, the La_2_Sr_2_PtO_7+δ_ exhibits a very small overpotential of 13 mV at a current density of −10 mA cm^−2^, close to that (3 mV) of commercial Pt black catalyst. The HER activity of La_2_Sr_2_PtO_7+δ_ was also assessed in H_2_-saturated 0.5 M H_2_SO_4_ to ensure the H_2_/H_2_O equilibrium for HER (Supplementary Fig. 6), which presents the superior HER activity (*η*_10_ of 27 mV and Tafel slope of 19 mV dec^−1^) among the state-of-the-art HER electrocatalysts (Supplementary Table 2). Besides, the stoichiometric La_2_Sr_2_PtO_7+δ_ oxide shows higher HER activity than other non-stoichiometric complex oxides (Supplementary Fig. 7), indicating that La_2_Sr_2_PtO_7+δ_ with stoichiometric element composition is the optimized catalyst for HER. To examine the kinetics and reaction mechanism, Tafel plots were drawn in Fig. 4b. The Tafel slope for La_2_Sr_2_PtO_7+δ_ (22 mV dec^−1^) is smaller than that for Pt black (30 mV dec^−1^), implying faster HER rates. Noticeably, such a small Tafel slop value of 22 mV dec^−1^ for La_2_Sr_2_PtO_7+δ_ suggests that the acidic HER electrocatalysis of the La_2_Sr_2_PtO_7+δ_ catalyst may follow a different reaction mechanism to conventional Volmer-Tafel, as will be discussed below. Above electrochemical analyses (e.g., small overpotential and low Tafel slope) highlight the extraordinary electrode activity of La_2_Sr_2_PtO_7+δ_ for HER in acidic media, although La_2_Sr_2_PtO_7+δ_ is one bulk material composed of micrometer-sized particles. Such excellent HER activity of La_2_Sr_2_PtO_7+δ_ is superior to that of reported HSBCCs and state-of-the-art Pt-based catalysts up to now (Fig. 4c and Supplementary Table 3), demonstrating that La_2_Sr_2_PtO_7+δ_ ranks the top HER electrocatalyst in acidic media. It’s known that two aspects (i.e., the intrinsic activity of each active site and the number of active sites) generally determine the overall catalytic activity of electrocatalysts^44^. To assess the intrinsic activity of La_2_Sr_2_PtO_7+δ_, we further calculated the specific activity by normalizing the electrode activity to the electrochemical surface area (ECSA) and real surface area (RSA). The values of ECSA and RAS of catalysts were estimated from the hydrogen underpotential deposition (H_UPD_) (Supplementary Fig. 8) and Brunner-Emmet-Teller measurements (Supplementary Fig. 9). Notably, the ECSA values of La_2_Sr_2_PtO_7+δ_ and Pt black were determined by integrating the charge of H_UPD_ desorption peak in cyclic voltammogram (CV) curves according to previous studies^45,46^. As we can see in Supplementary Fig. 8, the H_UPD_ integrated area of La_2_Sr_2_PtO_7+δ_ (1.99 m^2^/g_Pt_) is obviously smaller than that (70.11 m^2^/g_Pt_, similar with previous studies^45,46^) of Pt black, which may stem from the low oxide surface area (2.8 m^2^ g^−1^) and bulk morphology of La_2_Sr_2_PtO_7+δ_. Surprisingly, regardless of the electrode activity normalized to the ECSA or RSA, the La_2_Sr_2_PtO_7+δ_ catalyst offers a much higher specific activity than Pt black (Fig. 4d, e). For instance, at *η* = 0.05 V, the enhancement values in specific activity normalized to the ECSA and RSA for La_2_Sr_2_PtO_7+δ_ are up to about 18 and 2.2 times as compared with Pt black, indicative of its superior intrinsic activity toward acidic HER. Moreover, turnover frequency (TOF) values of La_2_Sr_2_PtO_7+δ_ and Pt black were calculated to further compare their intrinsic activity, which represents the amount of H_2_ molecule evolving per active site per second. TOF was plotted vs. potential (Fig. 4f) based on the calculated numbers of surface active sites according to the previously-reported methods (see Supplementary Fig. 10 and Supplementary Note. 1)^47,48^. Remarkably, the La_2_Sr_2_PtO_7+δ_ delivers an high TOF value of 596 s^−1^ at overpotential of 0.05 V, which is about two orders of magnitude higher than the commercial Pt black. Although La_2_Sr_2_PtO_7+δ_ possesses higher intrinsic activity than commercial Pt black, its mass activity is inferior to Pt black due to lower surface area (Supplementary Fig. 11); future ongoing investigation could shed more light on improving the surface area of La_2_Sr_2_PtO_7+δ_. As a control sample, a well-known Pt/WO_3_ HSBCC was also prepared for comparison (Supplementary Fig. 12a)^21^. As seen from Supplementary Fig. 12b, the single-component La_2_Sr_2_PtO_7+δ_ shows much higher intrinsic activity than binary-component Pt/WO_3_, confirming the superiority of the HSSCC system over the HSBCC system.

In addition to the catalytic activity, we also take the long-term durability of the La_2_Sr_2_PtO_7+δ_ catalyst into consideration to assess its potential for practical application. For this purpose, the accelerated durability tests (ADT) by continuous cycling within HER potential window were conducted. As shown in Fig. 4g, after 1000-cycling, the La_2_Sr_2_PtO_7+δ_ exhibits only slight activity decline but Pt black suffers from obvious activity loss. For example, La_2_Sr_2_PtO_7+δ_ displays a decay of only 10% whereas a nearly 5.4-fold faster decay rate (54%) was observed for Pt black during the period of continuous 1000-cycle operation (Fig. 4h, i). The poor stability of Pt black catalyst may be associated with the dissolution of Pt surface atoms and the agglomeration of Pt particles^49–51^. In conjunction with a series of post-HER measurements including XRD, XAS, TEM and STEM-EDS (Supplementary Fig. 13–16), some metallic Pt species are formed on the surface, which may account for slight activity decline after 1000-cycling due to inferior activity of metallic Pt than bulk La_2_Sr_2_PtO_7+δ_ component. According to the linear fitting of XANES, the average Pt valence of La_2_Sr_2_PtO_7+δ_ after HER is determined to be ~3.4 and thus the content of metallic Pt in post-HER La_2_Sr_2_PtO_7+δ_ is ~15% (Supplementary Fig. 17). Besides, the SEM image showing in Supplementary Fig. 18 indicates the nearly unchanged morphology of La_2_Sr_2_PtO_7+δ_ after HER. It should be noted that short-time cycling (e.g., 10-cycling) does not lead to the activity loss and Pt formation of La_2_Sr_2_PtO_7+δ_, as reflect by almost overlapped polarization curve with the initial one and the absence of metallic Pt peak in XRD pattern after 10-cycling. Besides, the negligible fluctuation of overpotential (@-10 mA cm^−2^) was observed for La_2_Sr_2_PtO_7+δ_ during 50 h chronopotentiometry test (Fig. 4j), which further confirms the robust operation durability for HER. Overall, the high electrode/intrinsic activity and electrochemical durability endow La_2_Sr_2_PtO_7+δ_ as a promising HER electrocatalyst candidate for future practical application in acidic water electrolysis.

### Experimental evidences for hydrogen spillover

**Fig. 5:**
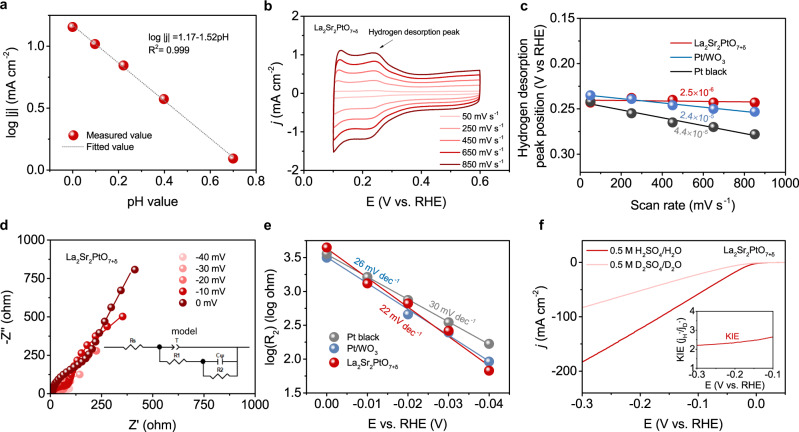
Experimental evidences for hydrogen spillover. **a** The Liner plot of log |*j* | at −0.03 V (vs. RHE) vs. pH. **b** CV profiles of La_2_Sr_2_PtO_7+δ_ catalyst with the scan rate from 50 to 850 mV s^−1^ in Ar-saturated 0.5 M H_2_SO_4_. **c** Plots of hydrogen desorption peak position vs. scan rates of the Pt black, Pt/WO_3_ and La_2_Sr_2_PtO_7+δ_ catalysts. **d** Nyquist plot for La_2_Sr_2_PtO_7+δ_ catalyst at various HER overpotentials. The scattered symbol represents the experimental results, and the solid lines is simulated fitting results. The inset also shows the equivalent circuit for the simulation. **e** EIS-derived Tafel plots of the Pt black, Pt/WO_3_ and La_2_Sr_2_PtO_7+δ_ catalysts obtained from the hydrogen adsorption resistance *R*_2_. **f** Polarization curves of La_2_Sr_2_PtO_7+δ_ catalyst in aqueous 0.5 M H_2_SO_4_ and 0.5 M D_2_SO_4_ solutions. The inset is the kinetic isotope effect value vs. potential.

In order to provide the evidences of possible atomic-scale hydrogen spillover in La_2_Sr_2_PtO_7+δ_ for acidic HER electrocatalysis, comprehensive experiments and electrochemical measurements were performed. The hydrogen spillover could be initially confirmed by the color change in the mixture of the catalyst and WO_3_^52^. As seen from Supplementary Fig. 19, The WO_3_ after HER test exhibits an unchanged color. However, the mixture of La_2_Sr_2_PtO_7+δ_ and WO_3_ after HER test generates a dark blue color, which is because that the spilled-over hydrogen migrates and readily reacts with WO_3_ to form dark blue H_x_WO_3_^52,53^. Besides, the hydrogen spillover effect could be also verified by Tafel slope and pH-dependent HER experiments. The Tafel slope of La_2_Sr_2_PtO_7+δ_ catalyst is only 22 mV dec^−1^, which is evidently lower than the value (30 mV dec^−1^) via conventional Volmer-Heyrovsky/Tafel mechanism, implying a hydrogen spillover-involving mechanism as also reported before^54–56^. This reaction mechanism was further supported by the pH-dependent relation of HER (Supplementary Fig. 20 and Fig. 5a). The reaction order of 1.52 for the La_2_Sr_2_PtO_7+δ_ catalyst is close to the theoretical value of 2 and similar with the reaction orders of the previously HSBCCs^54–56^.

In addition, referring to previous study, in situ analyzing the hydrogen adsorption and desorption kinetics on catalysts can support the occurrence of hydrogen spillover^57^. To examine the hydrogen desorption kinetics, the operando CV investigations were implemented and hydrogen desorption peaks during CV scanning in the double layer region were monitored^21,58^. For comparison, non-hydrogen-spillover metallic Pt black and well-known Pt/WO_3_ HSBCC samples were also studied. The CV curves of Pt black, Pt/WO_3_ and La_2_Sr_2_PtO_7+δ_ catalysts show the hydrogen desorption peak shift depending on the scan rate (Fig. 5b and Supplementary Fig. 21). Thus, it is rational to quantify their hydrogen desorption kinetics via plotting hydrogen desorption peak position vs. scan rate and comparing the fitted slopes. As shown in Fig. 5c, the slope follows the order of Pt black > Pt/WO_3_ > La_2_Sr_2_PtO_7+δ_. The significantly reduced slope for La_2_Sr_2_PtO_7+δ_ suggests its accelerated hydrogen desorption kinetics. It was reported that the hydrogen desorption kinetics for metal-support electrocatalysts could be effectively accelerated by hydrogen spillover effect^21,57^. Therefore, the fast kinetics of the hydrogen desorption for La_2_Sr_2_PtO_7+δ_ could be originated from the efficient hydrogen spillover. The operando electrochemical impedance spectroscopy (EIS) investigations were further carried out on Pt black, Pt/WO_3_ and La_2_Sr_2_PtO_7+δ_ catalysts at different overpotentials to investigate the hydrogen adsorption kinetics. The recorded Nyquist plots were simulated by a double-parallel equivalent circuit model (Fig. 5d, Supplementary Fig. 22 and Table 4). Following a previous recognition, the second parallel component *R*_2_ (*R*_2_ represents the hydrogen adsorption resistance) can reflect the hydrogen adsorption behavior on catalyst surface^24,57^. In view of the potential-dependent *R*_2_ for all catalysts, it is reasonable to quantify their hydrogen adsorption kinetics via plotting log*R*_2_ vs. overpotential and calculating the EIS-derived Tafel slopes by virtue of the Ohm’s law^57,59^. As displayed in Fig. 5e, the decreased slope for La_2_Sr_2_PtO_7+δ_ indicates an accelerated hydrogen adsorption kinetics, which may be associated with the promoted hydrogen spillover^57^.

Furthermore, H/D kinetic isotope effects (KIEs) can reflect the hydrogen or proton transfer kinetic information of chemical reactions, and the presence of KIEs (KIEs > 1.5) is considered as evidence that proton or hydrogen transfer is involved to affect the reaction rate^60–63^. The KIEs experiment in 0.5 M D_2_SO_4_/D_2_O solution was performed to obtain insight into the role of hydrogen transfer during the HER. The polarization curve of La_2_Sr_2_PtO_7+δ_ in 0.5 M D_2_SO_4_/D_2_O solution exhibits significantly lower current density in comparison with that of La_2_Sr_2_PtO_7+δ_ in the 0.5 M H_2_SO_4_/H_2_O solution by a factor about 2.2–2.6 (KIEs = 2.2–2.6) over the entire potential range (Fig. 5e). The KIEs value of La_2_Sr_2_PtO_7+δ_ suggests that the hydrogen or proton transfer involved in HER process is one possible step affecting the reaction rate.

Overall, the above experiments collectively verify the possible occurrence of hydrogen spillover in La_2_Sr_2_PtO_7+δ_. Currently, it’s very difficult to directly observe the electrocatalytic hydrogen spillover phenomenon, but we expect that in the future it could be resolved with the development of more advanced methodologies and technologies, such as transient imaging technology with super-high spatial resolution and nano-sized three-electrode electrochemical system.

### DFT calculations

**Fig. 6:**
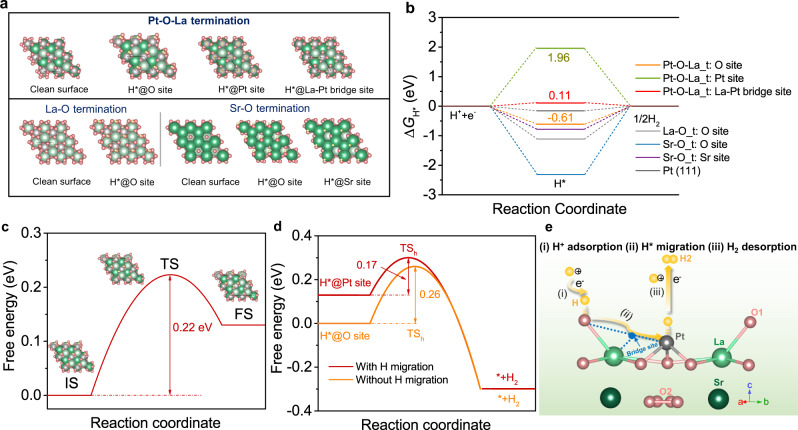
DFT calculations. **a** (001) surface slab models with different terminations of La_2_Sr_2_PtO_7+δ_ and the optimized structures. The yellow, red, gray, light and dark green balls represent H, O, Pt, La and Sr, respectively. **b** Gibbs free energy diagram for hydrogen adsorption at different catalytic sites on La_2_Sr_2_PtO_7+δ_ and Pt (111). **c** The kinetic energy barrier of hydrogen migration/spillover process for La_2_Sr_2_PtO_7+δ_. (IS the initial state, TS the transition state, FS the final state). **d** The free energy profiles of Heyrovsky step in HER on La_2_Sr_2_PtO_7+δ_ (with and without hydrogen migration/spillover). **e** Schematic illustration of catalysis mechanism for acidic HER via atomic-scale hydrogen spillover on the La_2_Sr_2_PtO_7+δ_ oxide.

To gain atomic-scale insight into the origin of the exceptional intrinsic activity of La_2_Sr_2_PtO_7+δ_ for HER in acid, density functional theory (DFT) calculations were carried out. Based on prior structural data and the analysis of HRTEM and SAED, (001) surface slab models with different terminations of La_2_Sr_2_PtO_7+δ_ and the optimized structures are shown in Fig. 6a and Supplementary Fig. 23. Generally, the acidic HER process involves a three-state diagram with an initial proton (H^+^), an intermediate adsorbed H* and a final H_2_ state, and the hydrogen adsorption Gibbs free energy (∆G_H*_) is taken as a commonly-accepted descriptor for accessing the intrinsic activity of electrocatalysts toward acid HER^64,65^. According to the Sabatier principle, the thermo-neutral active sites with an optimal  | ∆G_H*_ | value close to zero can facilely promote the adsorption and desorption process during HER^25,66^. Figure 6b shows the free energy diagram of La_2_Sr_2_PtO_7+δ_ on all the possible sites at the terminations of Pt-O-La, La-O and Sr-O along with Pt (111) metal as a reference. It is found that the H adsorption on the sites of La-O and Sr-O terminations is all too strong, which is not beneficial HER. On the Pt-O-La termination, the ΔG_H*_ values on O and Pt sites are −0.61 and 1.96 eV, respectively, indicating that the H adsorption is either too strong or too weak. Impressively, the calculated G_H*_ for the unique La-Pt bridge site is 0.11 eV, an optimal value close to a thermo-neutral state even exceeding that (−0.16 eV) for the state-of-art Pt (111) surface, suggesting the hydrogen adsorption is neither too strong nor too weak. Thus, the Pt-O-La termination of La_2_Sr_2_PtO_7+δ_ with three different H adsorption sites is chosen for studying the HER process.

As reported before, hydrogen spillover has been an efficient strategy to boost the intrinsic HER activity of the binary-component electrocatalysts^21–24^. These HSBCCs generally consist of one hydrogen-enriched component with a negative ∆G_H*_ value and one hydrogen-poor component with a positive ∆G_H*_ value, and hydrogen spillover takes place from the surface with ∆G_H-negative_ to the surface with ∆G_H-positive_^23,24^. When a single-component catalyst contains more than one kind of catalytic site, the hydrogen spillover effect between different sites may also happen and can affect the overall catalytic activity. With regard to single-component La_2_Sr_2_PtO_7+δ_ catalyst, the H^+^ preferentially adsorbs at O site among three possible sites (i.e., O site, La-Pt bridge site and Pt site) due to the negative ΔG_H*_ value of −0.61 eV, suggesting significant proton trapping at O site which functions as hydrogen-enriched “component”. Conversely, the H exhibits weak adsorption on the Pt site with ΔG_H*_ value of 1.96 eV, indicating Pt site with high oxidation state resembles the hydrogen-poor “component” and is advantageous for H_2_ desorption. More importantly, the thermo-neutral La-Pt bridge site can serve as the mediator for favorable hydrogen spillover, similar to the interface site in the conventional HSBCCs. In view of the steadily weakened H adsorption, a feasible channel for the hydrogen spillover from O site → La-Pt bridge site → Pt site may be formed on the La_2_Sr_2_PtO_7+δ_ catalyst. Noticeably, the interface-free feature and short reaction distance within the crystal lattice on single-phase La_2_Sr_2_PtO_7+δ_ catalyst is beneficial for minimizing the kinetic barrier for hydrogen spillover^52,67^, somewhat analogous to the reported metal alloy system with low hydrogen spillover barrier^68–71^. The calculation shows that the hydrogen spillover step has a low energy barrier value of 0.22 eV (Fig. 6c and Supplementary Fig. 24), indicating that the H migration from O site to Pt site is a kinetically favorable process. Then, we calculated the energy barriers of Heyrovsky step with and without hydrogen spillover. As seen from Fig. 6d, the energy barrier on Pt site after H migration is 0.17 eV, which is lower than that (0.26 eV) on O site without H migration, demonstrating the superiority of hydrogen spillover/migration of La_2_Sr_2_PtO_7+δ_ for HER catalysis. It should be also noted that the theoretical overpotential (0.22 V) of La_2_Sr_2_PtO_7+δ_ for the whole HER process involving the hydrogen spillover is lower than that (0.69 V) for the conventional HER mechanism on Pt surface^72^. Based on these DFT results, a possible HER catalytic mechanism on La_2_Sr_2_PtO_7+δ_ in acidic media via the synergy of multi-function catalytic sites was proposed, as schematically illustrated in Fig. 6e. Namely, the O site serves as the proton enrichment, the thermo-neutral La-Pt bridge site serves as the mediator for favorable hydrogen spillover/migration from O site to Pt site, and Pt site functions the final H_2_ desorption. In order to further confirm the active sites of La_2_Sr_2_PtO_7+δ_ for HER, poisoning tests was performed by adding thiocyanate (SCN^−^) and tetramethylammonium cation (TMA^+^) ions into acidic solutions (Supplementary Fig. 25)^73,74^. The La_2_Sr_2_PtO_7+δ_ exhibits evident performance decay after adding SCN^-^ or TMA^+^ into H_2_SO_4_ solution, indicating that the Pt and O ions are both active sites of La_2_Sr_2_PtO_7+δ_ for HER. However, Pt/WO_3_ shows evident activity loss after adding SCN^-^ and has nearly no activity loss after adding TMA^+^, implying that the metallic Pt is the sole active site. Such result demonstrates the direct actives of Pt and O ions for HER in La_2_Sr_2_PtO_7+δ_, which is initiated by the hydrogen spillover/migration and accordingly contributes to the high performance.

## Discussion

In summary, we have successfully synthesized a single-phase complex oxide La_2_Sr_2_PtO_7+δ_ with exceptional HER performance in acid medium via a facile and scalable solid-state reaction method. The La_2_Sr_2_PtO_7+δ_ displays an excellent HER activity with an ultralow overpotential of 13 mV at a current density of 10 mA cm^−2^ and a small Tafel slope of 22 mV dec^−1^ in 0.5 M H_2_SO_4_ solution, surpassing state-of-the-art Pt-based catalysts and HSBCC ever reported. Besides, significant intrinsic activity and durability enhancement were observed for La_2_Sr_2_PtO_7+δ_ relative to the commercial Pt black. By coupling DFT simulations and comprehensive experiments, the high HER catalytic activity of La_2_Sr_2_PtO_7+δ_ in acid possibly results from an unusual atomic-scale hydrogen spillover effect between multiple catalytic sites, whereby O site captures proton, H facilely diffuses from O site to Pt site with thermoneutral La-Pt bridge site serving as the mediator, and eventually as-formed H_2_ favorably releases on Pt site. Our proof-of-concept investigations not only provides the atomic-level insight into the hydrogen spillover within La_2_Sr_2_PtO_7+δ_ for acidic HER, but also open a new avenue for the design of advanced electrocatalysts via constructing multifunctional catalytic sites.

## Methods

### Catalyst synthesis

La_2_Sr_2_PtO_7+δ_ catalyst was synthesized via the traditional solid-phase reaction method. Firstly, stoichiometric amounts of La_2_O_3_, SrCO_3_, and Pt were weighed and mixed in ethanol and water under the rotation speed of 400 rpm for 1 h through the high-energy ball-milling (Planetary Mono Mill, Pulverisette 6, Fritsch). Then the homogeneously dispersed mixture was dried and finally calcined at 1100 °C in air for 10 h to obtain the resultant catalyst powders. Pt/WO_3_ complex catalyst was prepared via the high-energy ball-milling method. In detail, WO_3_ and Pt were weighed at mass ratio of 75:25 and mixed in ethanol and water under the rotation speed of 400 rpm for 1 h through the high-energy ball-milling. The homogeneously dispersed mixture was finally dried to obtain the resultant catalyst powders.

### Characterizations

XRD patterns were measured using a Rigaku Smartlab diffractometer operating at 40 kV with filtered Cu Kα radiation. The Rietveld refinements were revealed using DIFFRAC plus Topas 4.2 software. SEM images were recorded through a scanning electron microscope equipped with the scanning electron microanalyzer (Hitachi S-4800). The HRTEM images were obtained utilizing the electron microscope (FEI Tecnai G2 F20) operating at 200 kV. STEM image and elemental mapping images were obtained using Tecnai F20 SuperTwin operating at 200 kV. Nitrogen adsorption-desorption isotherms were recorded on BELSORP II. Pt4f spectra were acquired on XPS (Perkin Elmer PHI 1600 ECSA system). X-ray absorption near-edge structure (XANES) and the EXAFS spectra were determined at the BL 07A beamline of the National Synchrotron Radiation Research Center in Taiwan. All samples were pretreated via cutting pellets in an ultrahigh vacuum chamber to obtain a clean surface. Hydrogen temperature-programmed desorption (H_2_-TPD) experiments were performed on Chembet Pulsar (Quantachrome Instruments, USA). In total, 50 mg of sample was pretreated at 200 °C for 3 h in hydrogen atmosphere and then was cleaned with argon gas flow at 50 °C for 30 min to remove weakly adsorbed H_2_. TPD process was performed by heating the sample from 50 to 600 °C at a ramp rate of 2.5 °C min^−1^ under argon atmosphere.

### Electrochemical measurements

HER measurements in acid media were conducted in a standard three-electrode electrochemical cell (Pine Research Instrumentation) in an RDE configuration using a CHI 760E electrochemistry workstation. Catalysts cast on RDE (5 mm in diameter), graphite rod, and Ag|AgCl (3.5 M KCl) were used as the working electrode, counter electrode, and reference electrode, respectively. Working electrodes for HER measurements were prepared by a controlled drop-casting method, in accordance with the previous works^2,64^. The mass loading of oxide catalysts and Pt black on the RDE is ~0.232 and 0.058 mg cm^−2^, respectively. Linear sweep voltammetry was recorded at 5 mV s^−1^ at the rotation of 2400 rpm in Ar or H_2_-saturated 0.5 M H_2_SO_4_. Tafel slopes were determined by plotting the overpotential vs. the logarithm of current density (log | *j* | ). CV curves involving in the H_upd_ adsorption/desorption peak were obtained in N_2_-saturated 0.5 M H_2_SO_4_ solution with potential window between 0.05 V and 1.1 V vs. RHE and a sweep rate of 100 mV s^−1^. The ECSA was derived from the H_upd_ desorption peak (0.03–0.35 V) area normalized by the total mass of the Pt element. ADT of catalysts was conducted through continuous potential cycling ranged from 0 to −0.4 V vs. Ag|AgCl for 1000 cycles at a scan rate of 100 mV s^−1^. The chronopotentiometry tests were performed at a constant cathodic current density of 10 mA cm^−2^ to explore the durability of the electrocatalysts. EIS measurements were carried out on the Bio-Logic SP-300 workstation at RT in the frequency range of 100 kHz–0.1 Hz at various HER overpotentials.

### Computational methods

All first-principle calculations were performed using DFT implemented in the Vienna Ab initio Simulation Package. The projector augmented wave pseudo potentials with the Perdew-Burke-Enrnzerhof functional were used. The cutoff of kinetic energy was set as 500 eV. La_2_Sr_2_PtO_7_ bulk with R-3 space group was built for lattice structure optimization. Monkhorst-Pack 7 × 7 × 2 *k*-point mesh was used during the structure relaxation. (001) surface slab models with different terminations were cleaved from the optimized bulk lattice to investigate H adsorption on surface. A 15Å-thick vacuum layer was added to the surface in the *z* direction. In total, 2 × 2 supercells were built to simulate HER process on (001) surface with La-O-Pt termination. Half layers on the bottom were fixed to mimic bulk arrangements, while other layers were fully relaxed to represent surface features. The first Brillouin zone of the slab model was samples with a 5 × 5 × 1 *k*-point grid. The first Brillouin zone of the slab model and supercell was sampled with a 5 × 5 × 1 and a 3 × 3 × 1 *k*-point grid, respectively. Transition states were determined using climbing image nudge elastic band method with 8 images. All calculations were performed in a spin-polarized fashion. The force and energy convergence criteria were set to 0.02 eV Å^−1^ and 10^−5^ e Å, respectively. Zero-point energy and entropy corrections were introduced for Gibbs energy calculations.

### Reporting summary

Further information on research design is available in the Nature Research Reporting Summary linked to this article.

## Supplementary information

Supplementary Information

Reporting Summary

## Data Availability

The data that support the findings of this study are available in the Source Data. Source data are provided with this paper.

## Source data

Source Data
