# Supplementary material for: Hydrogen spillover in complex oxide multifunctional sites improves acidic hydrogen evolution electrocatalysis
Source: Nat Commun. 2022 Mar 4;13:1189. doi: 10.1038/s41467-022-28843-2 (PMC8897394; doi:10.1038/s41467-022-28843-2)
Supplement: Supplementary file 1 — Supplementary Information [file 41467_2022_28843_MOESM1_ESM.pdf]

Supplementary Information

Hydrogen spillover in complex oxide multifunctional sites improves acidic hydrogen evolution electrocatalysis

Dai et al.

Supplementary Figures

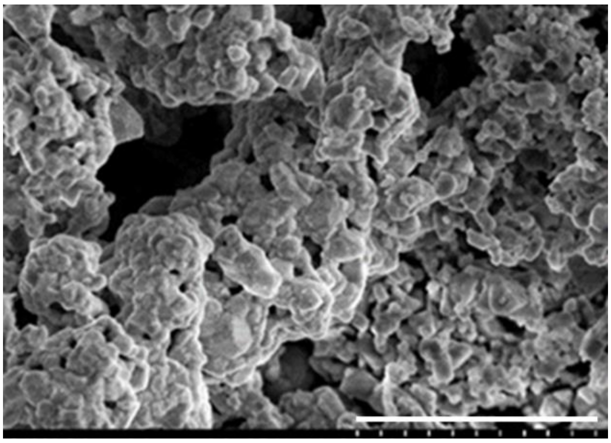

Supplementary Figure 1. Morphology characterization of $La_2Sr_2PtO_{7+\delta}$. SEM image of $La_2Sr_2PtO_{7+\delta}$. The scale bar is 5 μm.

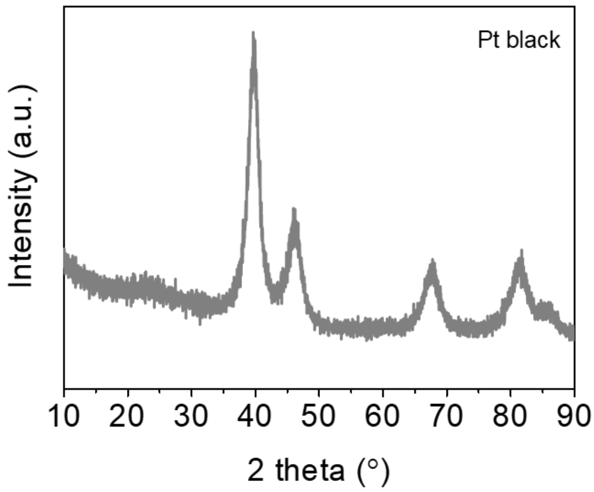

Supplementary Figure 2. Crystal structure characterization of Pt black. XRD pattern of Pt black.

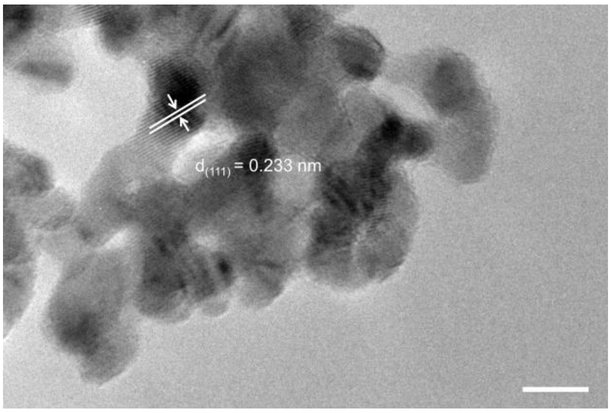

Supplementary Figure 3. HRTEM image of Pt black. The particle size of Pt black is about 5nm. The lattice spacing is about 0.233 nm for (111) plane. The scale bar is 5 nm.

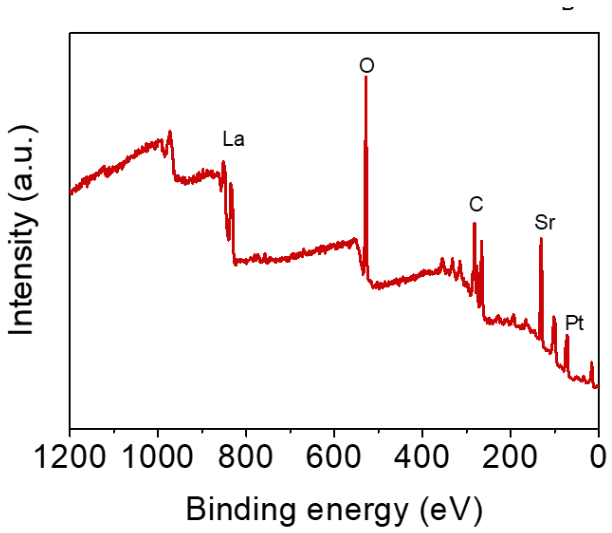

Supplementary Figure 4. XPS survey of $La_2Sr_2PtO_{7+\delta}$. XPS survey of $La_2Sr_2PtO_{7+\delta}$ confirms the coexistence of La, Sr, Pt, O elements.

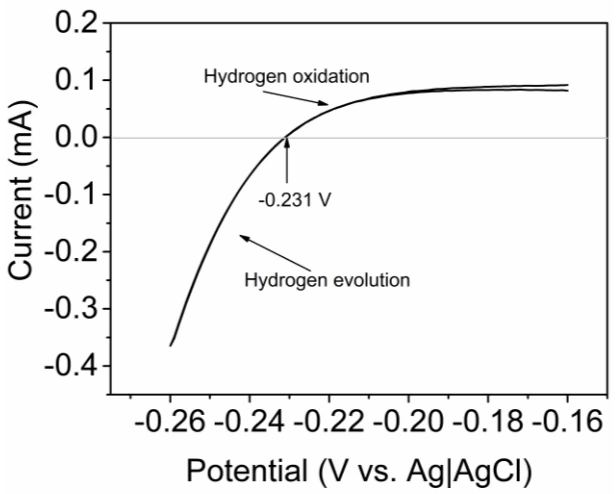

Supplementary Figure 5. RHE calibration. RHE calibration of the Ag|AgCl reference electrode in 0.5 M H_2SO_4. The calibration process was performed in a high purity H_2-saturated 0.5 M H_2SO_4 with a platinum RDE (PINE, 0.126 cm^2) as the working electrode, Pt foil as the counter electrode, and Ag|AgCl (3.5 M KCl) as the reference electrode. Cyclic voltammetry (CV) was conducted at a scan rate of 1 mV s^{-1}, and the average of the two potentials at which the current crossed zero was taken as the thermodynamic potential for the hydrogen electrode reaction. In 0.5 M H_2SO_4 solution, $E_{RHE} = E_{Ag|AgCl} + 0.231$ V.

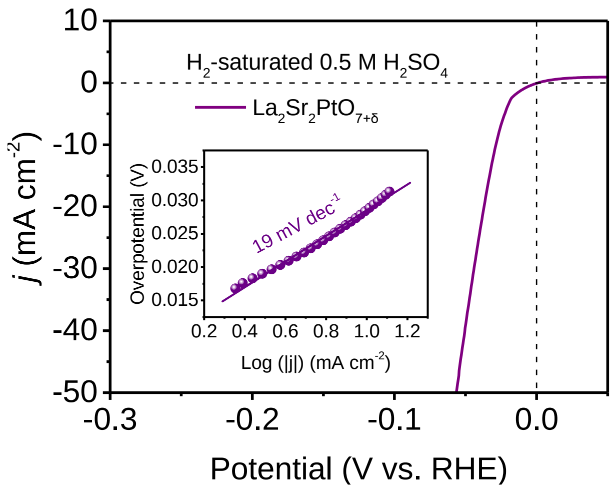

Supplementary Figure 6. HER performance of the $La_2Sr_2PtO_{7+\delta}$ catalysts in H_2-saturated 0.5 M H_2SO_4 solution. HER polarization curve of the $La_2Sr_2PtO_{7+\delta}$ catalysts in H_2-saturated 0.5 M H_2SO_4 solution. Inset is the corresponding Tafel plot.

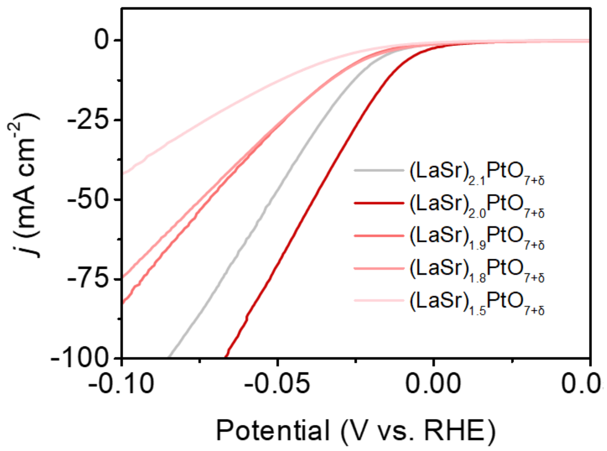

Supplementary Figure 7. HER activity of $(LaSr)_{2-x}PtO_{7+\delta}$ (x=-0.1, 0, 0.1, 0.2, 0.5). HER polarization curve of $La_2Sr_2PtO_{7+\delta}$ and non-stoichiometric complex oxides, including A-site-rich $(LaSr)_{2.1}PtO_{7+\delta}$ and A-site-deficient $(LaSr)_{1.9}PtO_{7+\delta}$, $(LaSr)_{1.8}PtO_{7+\delta}$, $(LaSr)_{1.5}PtO_{7+\delta}$.

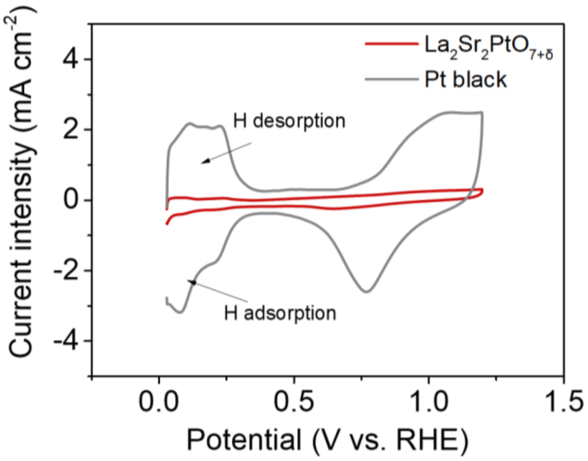

Supplementary Figure 8. Cyclic voltammetry curves for determining the H_{upd} desorption peak area. Cyclic voltammetry curves of $La_2Sr_2PtO_{7+\delta}$ and Pt black.

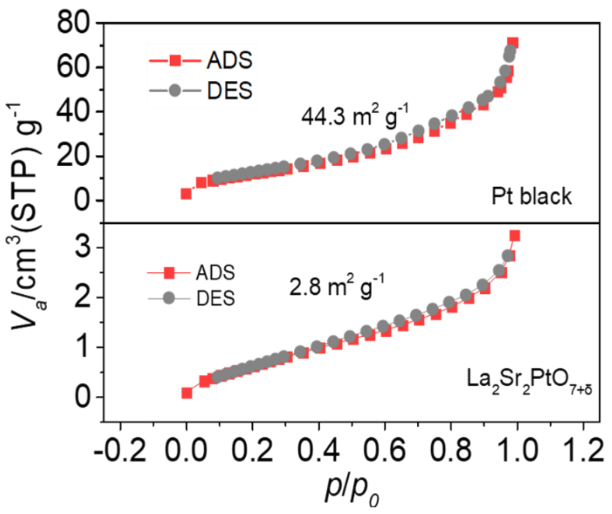

Supplementary Figure 9. N_2 adsorption-desorption isotherm of Pt black and $La_2Sr_2PtO_{7+\delta}$. The calculated specific surface area in BET method of Pt black and $La_2Sr_2PtO_{7+\delta}$ is 44.3 and 2.8 m^2 g^{-1}, respectively.

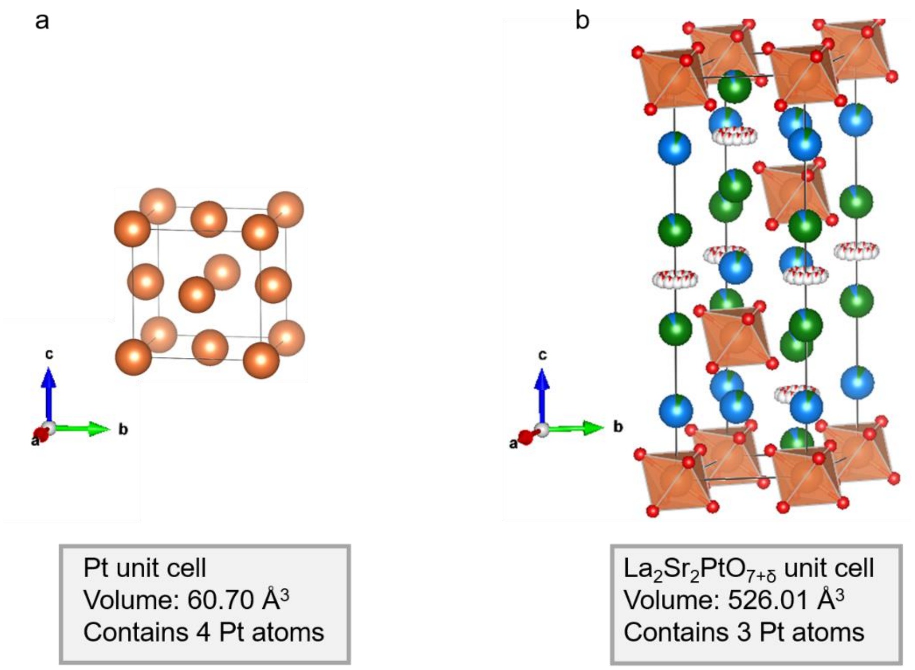

Supplementary Figure 10. Unit cell of catalysts. (a) Pt black and (b) $La_2Sr_2PtO_{7+\delta}$.

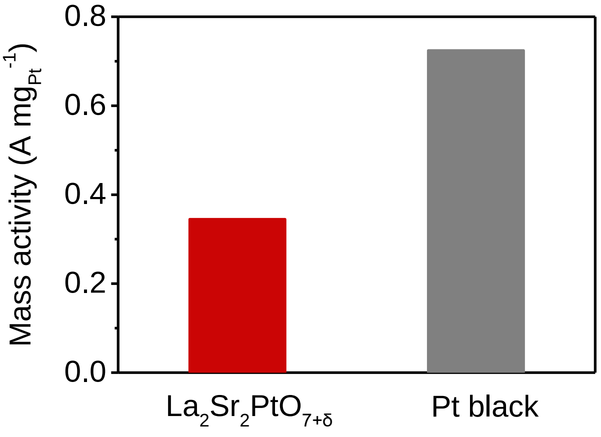

Supplementary Figure 11. Mass activity comparison between Pt black and $La_2Sr_2PtO_{7+\delta}$. Mass activity of Pt black and $La_2Sr_2PtO_{7+\delta}$ at -0.02 V.

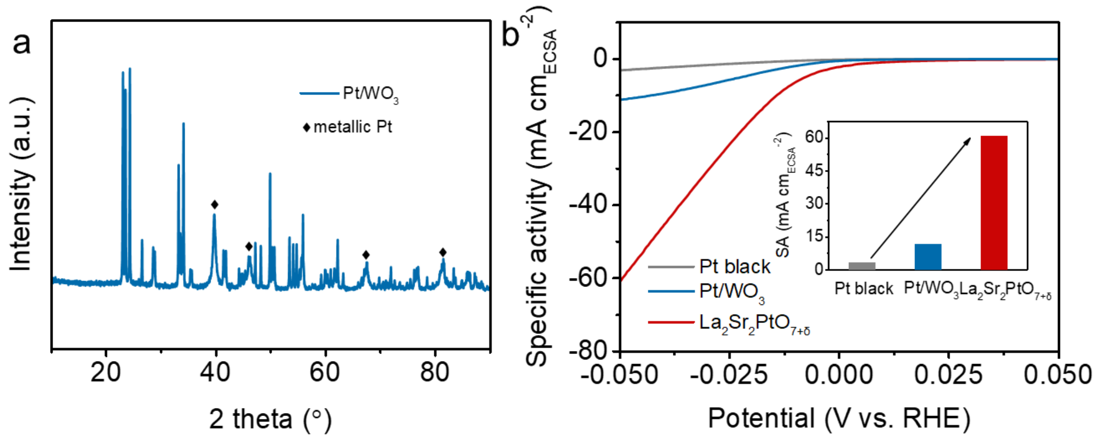

Supplementary Figure 12. Structural and electrochemical characterization of Pt/WO_3. (a) XRD of Pt/WO_3. (b) Specific activity normalized to ECSA of Pt/WO_3 as a function of applied potential. Inset: specific activity at the overpotential of η = 0.05 V.

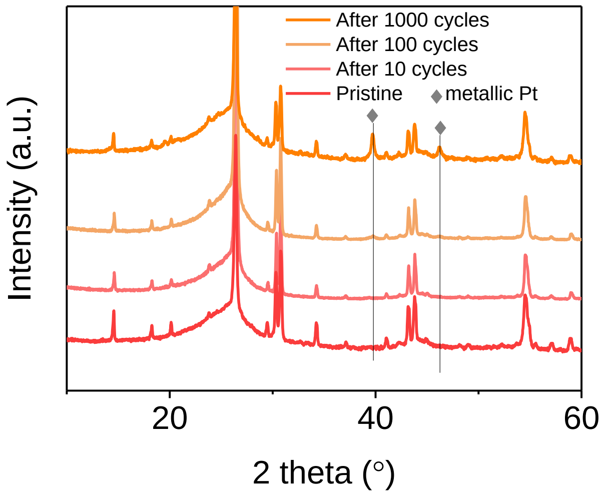

Supplementary Figure 13. Structural change of $La_2Sr_2PtO_{7+\delta}$ during HER. XRD patterns of $La_2Sr_2PtO_{7+\delta}$ before and after different CV cycles during HER.

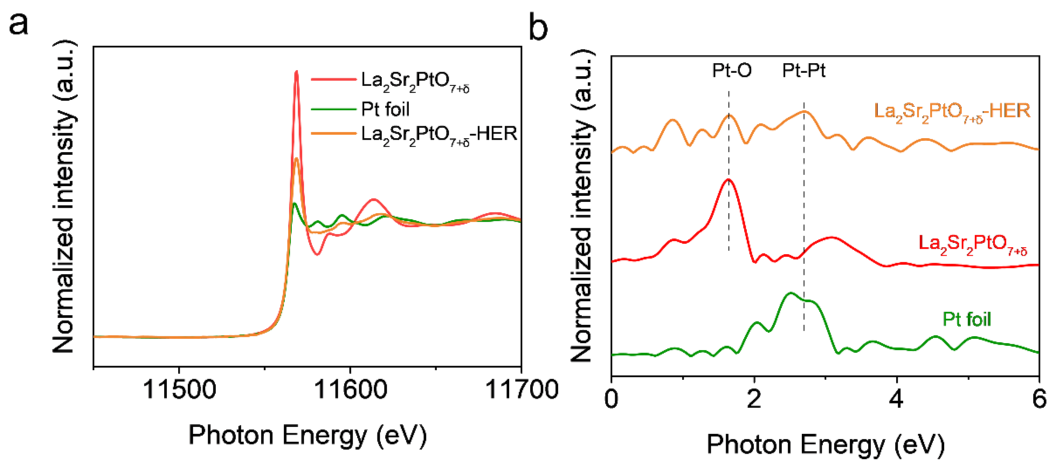

Supplementary Figure 14. Chemical state and coordination environment change of $La_2Sr_2PtO_{7+\delta}$ during HER. (a) Pt L_3-edge XANES spectra of $La_2Sr_2PtO_{7+\delta}$ before and after 1000 cycles during HER as well as Pt foil. (b) K^3-weighted Fourier transform EXAFS spectra of the $La_2Sr_2PtO_{7+\delta}$ before and after 1000 cycles during HER as well as Pt foil.

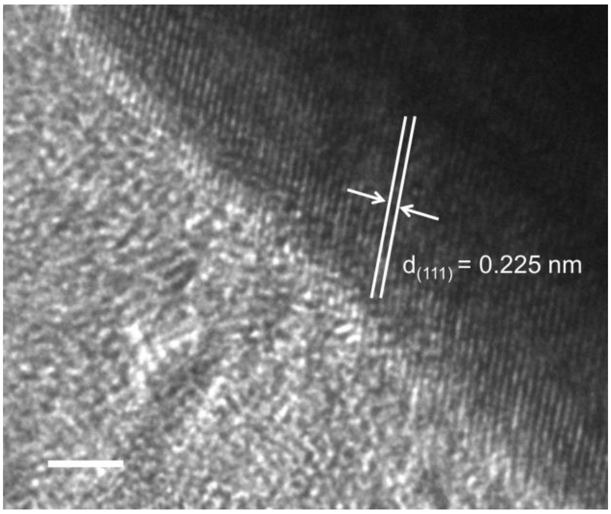

Supplementary Figure 15. HRTEM image of post-ADT $La_2Sr_2PtO_{7+\delta}$. The scale bar is 2 nm.

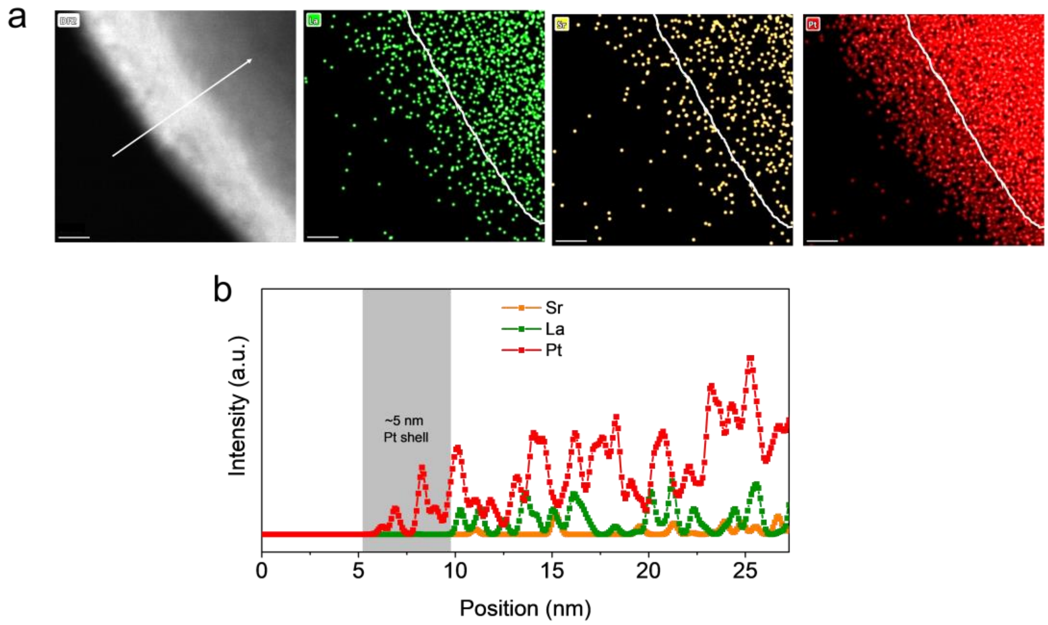

Supplementary Figure 16. Surface element distribution change during HER. (a) HAADF-STEM and the corresponding elemental mapping images of post-ADT $La_2Sr_2PtO_{7+\delta}$. The scale bar is 5 nm. (b) STEM-EDS spectrum of post-ADT $La_2Sr_2PtO_{7+\delta}$.

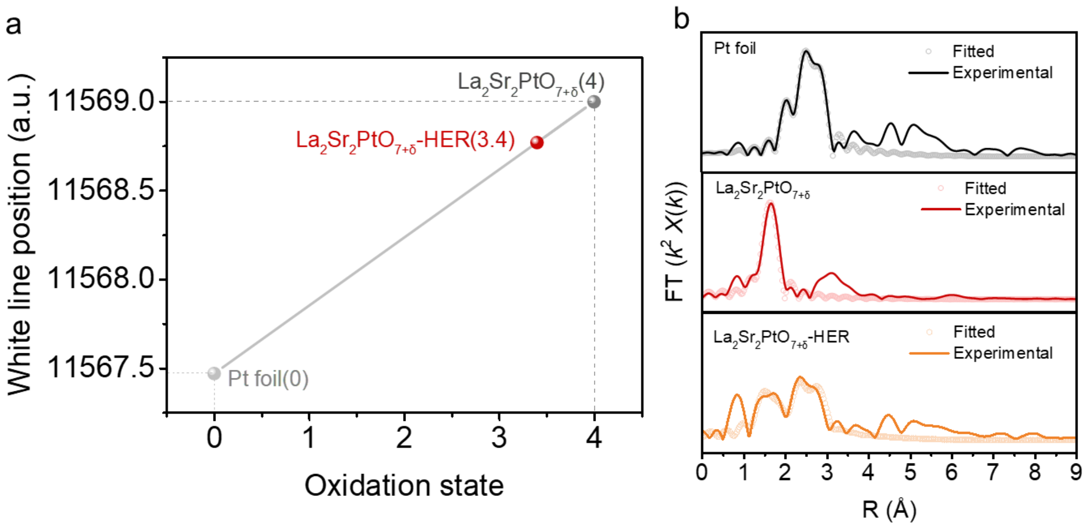

Supplementary Figure 17. The fitted average oxidation states and coordination environment of Pt in $La_2Sr_2PtO_{7+\delta}$. (a) The fitted average oxidation states of Pt in $La_2Sr_2PtO_{7+\delta}$ from XANES spectra; (b) First-shell fitting of EXAFS spectra of the Pt foil, $La_2Sr_2PtO_{7+\delta}$ and $La_2Sr_2PtO_{7+\delta}$-HER catalysts.

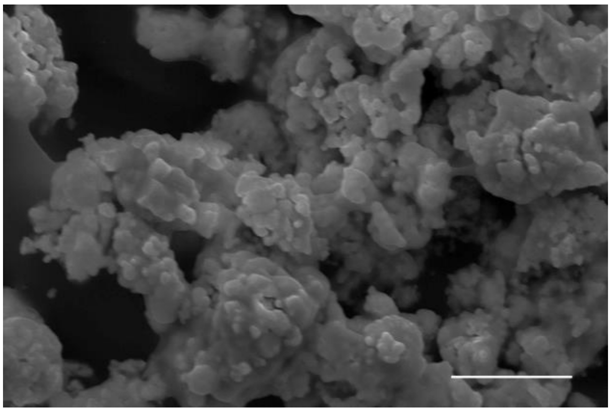

Supplementary Figure 18. SEM image of $La_2Sr_2PtO_{7+\delta}$ after HER. The scale bar is 10 μm.

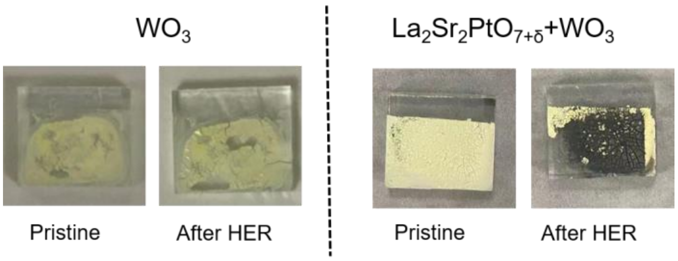

Supplementary Figure 19. Color change photographs of the mixtures. Pictures at left are pristine WO_3 film and WO_3 film after HER on indium tin oxide (ITO) electrode. Pictures at right are pristine mixture film and mixture film after HER on ITO electrode.

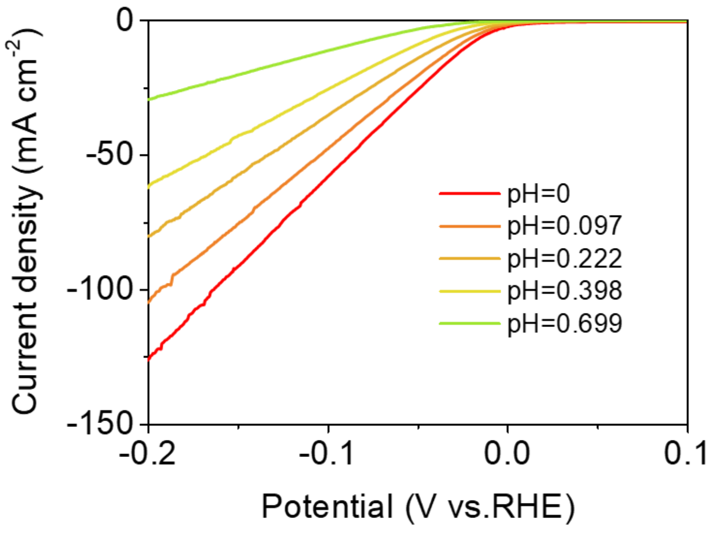

Supplementary Figure 20. The pH-dependance measurements. LSV curves of $La_2Sr_2PtO_{7+\delta}$ in Ar-saturated H_2SO_4 electrolyte with pH ranging from 0 to 0.699.

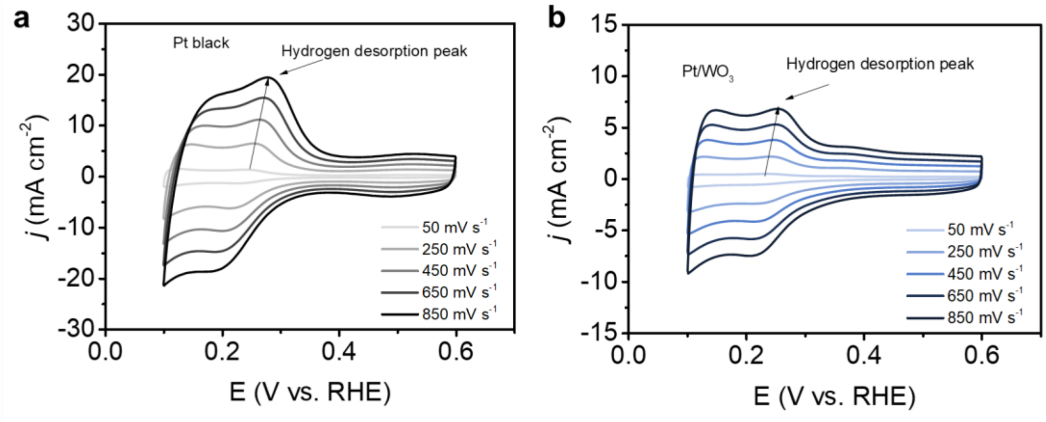

Supplementary Figure 21. Hydrogen desorption peak shift depending on the scan rate of Pt black and Pt/WO_3 catalyst. CV profiles of (a) Pt black and (b) Pt/WO_3 catalyst with the scan rate from 65 to 850 mV s^{-1} in Ar-saturated 0.5 M H_2SO_4.

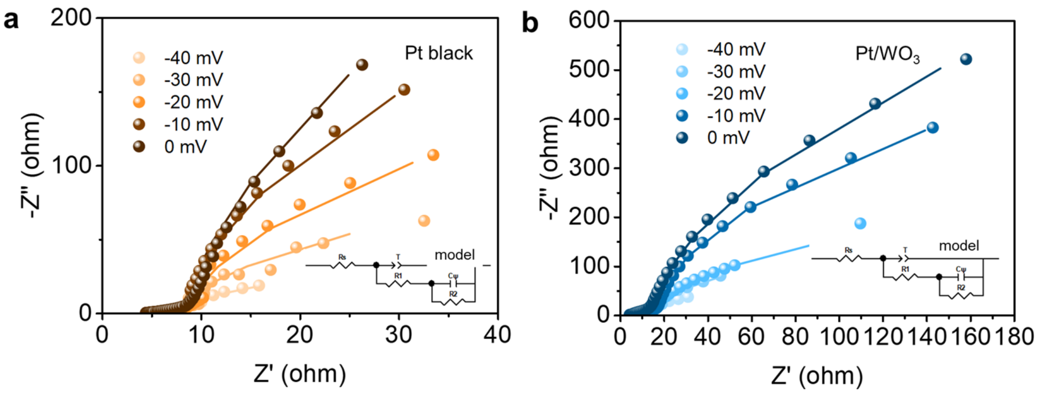

Supplementary Figure 22. The *operando* electrochemical impedance spectroscopy (EIS) investigations of Pt black and Pt/WO_3 catalyst. Nyquist plot for (a) Pt black and (b) Pt/WO_3 catalyst at various HER overpotentials. The scattered symbol represents the experimental results, and the solid lines is simulated fitting results. The inset also shows the equivalent circuit for the simulation.

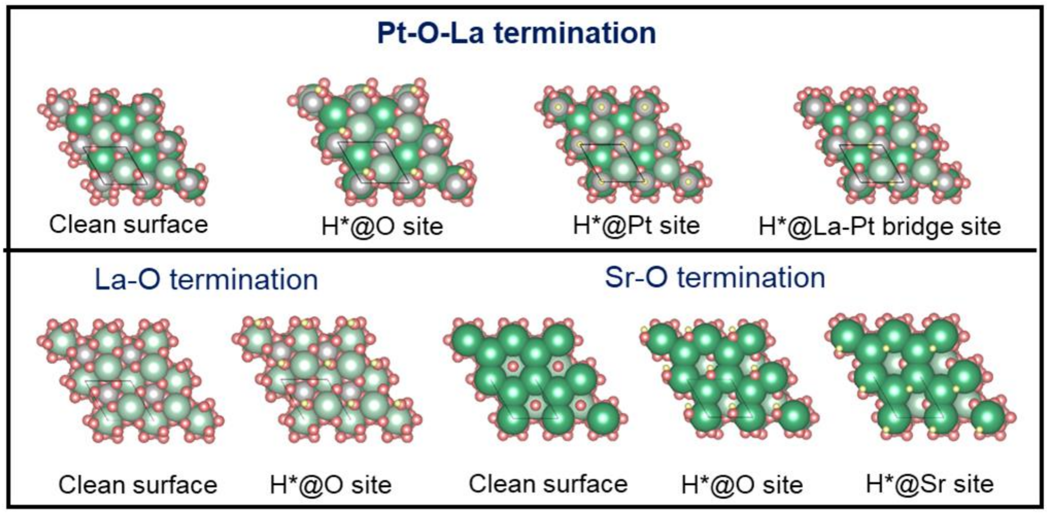

Supplementary Figure 23. The enlarged (001) surface slab models and the optimized structures. The (001) surface slab models with different terminations (Pt-O-La, La-O and Sr-O) of $La_2Sr_2PtO_{7+\delta}$ and the optimized structures. The yellow, red, gray, light and dark green balls represent H, O, Pt, La and Sr, respectively.

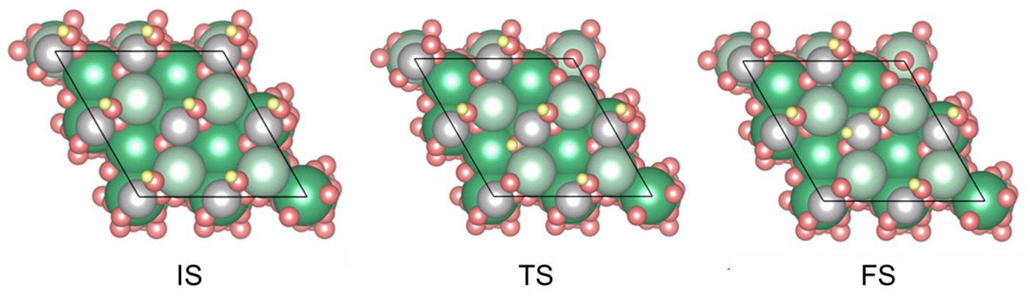

Supplementary Figure 24. The enlarged slab models of different state during hydrogen migration/spillover process of $La_2Sr_2PtO_{7+\delta}$. The enlarged slab models of the initial state (TS), the transition state (TS), and the final state (FS) for hydrogen migration/spillover process of $La_2Sr_2PtO_{7+\delta}$.

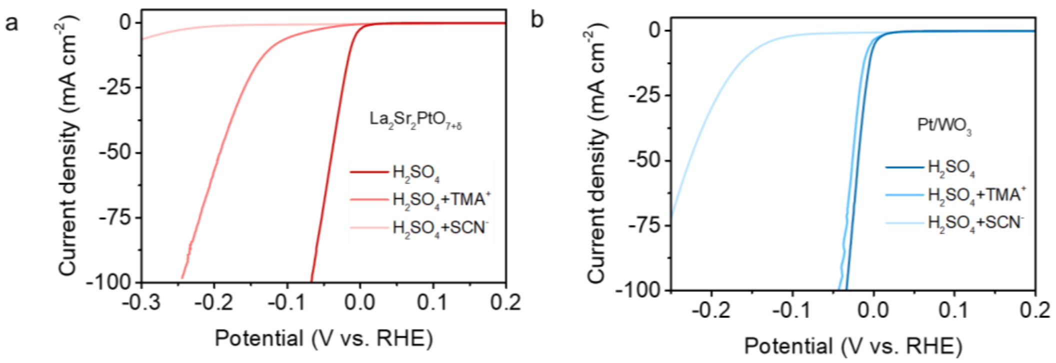

Supplementary Figure 25. Poisoning tests. Polarization curves for (a) $La_2Sr_2PtO_{7+\delta}$ and (b) Pt/WO_3 before and after the addition of SCN^- and TMA^+ ions in Ar-saturated 0.5 M H_2SO_4.

Supplementary Tables

Supplementary Table 1. Atomic coordinates, isotropic displacement parameters and cell parameters for $La_2Sr_2PtO_{7+\delta}$.

Site	Np	x	y	z	Occ
Pt	3a	0	0	0	1
La1	6c	0	0	0.6233	0.915
Sr1	6c	0	0	0.6233	0.085
La2	6c	0	0	0.1731	0.085
Sr2	6c	0	0	0.1731	0.915
O1	18f	0.3145	0.2169	0.0621	1
O2	18f	0.1258	0	0.5	0.082
O3	18f	0.1452	0.0726	0.5	0.082

Space group: $R\overline{3}$, a=b=5.7913(2) Å, c=18.1097(7) Å, V= 526.0149(5) Å^3, R_{wp} = 8.85,R_p = 6.80, χ^2 = 1.40.

Supplementary Table 2. Comparison of HER activity in H_2-saturated 0.5 M H_2SO_4 solution for $La_2Sr_2PtO_{7+\delta}$ with the state-of-the-art HER electrocatalysts, especially Pt-based catalysts

**Catalysts**	**η_{10} (mV)**	**Tafel slope (mV dec^{-1})**	**Mass loading (mg·cm^{-2})**	**TOF (s^{-1})**	**Refs**
$La_2Sr_2PtO_{7+\delta}$	27	19	0.232	1303 @-0.1 V	This work
Pt SA/m-WO_{3-x}	47	45	0.196	35 @-0.1 V	*Angew. Chem. Int. Edit.* 58, 16038-16042, (2019).
Pt_1/OLC	38	36	0.510	41 @-0.1 V	*Nat. Energy* 4, 512-518 (2019).
$Mo_2TiC_2T_x$-Pt_{SA}	30	30	1	N.A.	*Nat. Catal.* 1, 985-992 (2018).
Pt-MoS_2	35	25	0.07	N.A.	*Nat. Commun.* 8, 1-9 (2017).
Pt-WO_3	42	33	0.141	3 @-0.08 V	*Nano Energy* 71, 104653 (2020).
RuP_2@NPC	38	38	1	N.A.	*Angew. Chem. Int. Edit.* 56, 11559-11564 (2017).

N.A.=Not available

Supplementary Table 3. HER activity comparison between $La_2Sr_2PtO_{7+\delta}$ and reported hydrogen spillover-based binary-component catalysts (HSBCCs) and state-of-the-art Pt-based catalysts in 0.5 M H_2SO_4.

**Catalysts**	**Substrate**	**Mass loading ($mg \cdot cm^{-2}$)**	***η* @10 mA cm^{-2} (mV)**	**Tafel slope (mV dec^{-1})**	**TOF (s^{-1})**	**Ref**
**$La_2Sr_2PtO_{7+\delta}$**	**Glass carbon**	**0.232**	**13**	**22**	**1303** @-0.1 V	**This work**
**Pt-group metal based catalysts**						
$Pt@C_2N$	Glass carbon	0.285	~40	33	N.A.	*Nat. Nanotech.*, 2017, 12, 441
PtRu@RFCS	Glass carbon	0.354	20	27.2	4 @-0.1 V	*Energy Environ. Sci.*, 2018, 11, 1232
Pt_1/N-C	Glass carbon	0.250	19	14	22 @-0.05 V	*Nat. Commun.*, 2020, 11, 1029
$Mo_2TiC_2T_x$-Pt_{SA}	Glass carbon	1	30	30	N.A.	*Nat. Catal.*, 2018, 1, 985
Pt@PCM	Glass carbon	N.A.	105	64	N.A.	*Sci. Adv.*, 2016, 2, e1501602
Pt/np-$Co_{0.85}Se$	Glass carbon	2.5	55	35	4 @-0.1 V	*Nat. Commun.*, 2019, 10, 1743
Pd/Cu-Pt NRs	Glass carbon	0.2	23	25	N.A.	*Angew. Chem. Int. Ed.*, 2017, 56, 16047
ALD50Pt/NGNs	Glass carbon	0.077	~38	29	N.A.	*Nat. Commun.*, 2016, 7, 13638
Pt_1/OLC	Glass carbon	0.510	38	35	41 @-0.1 V	*Nat. Energy*, 2019, 4, 512
Pt-GT-1	Glass carbon	0.280	18	24	7 @-0.07 V	*Nat. Energy*, 2018, 3, 773
PtNC/S-C	Glass carbon	0.051	11	24	N.A.	*Nat. Commun.*, 2019, 10, 4977
PtW_6O_{24}/C	Glass carbon	N.A.	22	30	33 @-0.1 V	*Nat. Commun.*, 2020, 11, 490
**Hydrogen spillover-based binary-component catalysts (HSBCCs)**						
Rh/SiNW	Glass carbon	0.193	~80	24	N.A.	*Nat. Commun.*, 2016, 7, 12272
Rh-MoS_2	Glass carbon	0.309	47	24	N.A.	*Adv. Funct. Mater.*, 2017, 27, 1700359
Ir/SiNW	Glass carbon	0.339	20	22	N.A.	*ACS Nano*, 2019, 13, 2786
Pt/CoP	Glass carbon	0.102	21	43	N.A.	*Energy Environ. Sci.*, 2019, 12, 2298
Pt-SA/ML-WO_3	Glass carbon	0.56	22	27	>250 @-0.1 V	*Adv. Funct. Mater.*, 2021, 2009770
Pt-WO_3	Glass carbon	0.141	39	33	3 @-0.08 V	*Nano Energy*, 2020, 71, 104653
Pt SA/m-WO_{3-x}	Glass carbon	0.196	47	45	35 @-0.1 V	*Angew. Chem. Int. Ed.*, 2019, 58, 16038
Pt/RuCeO_x-	Glass	0.388	45	31	N.A.	*Angew. Chem.*

PA	carbon					*Int. Ed.*, 2020, 59, 20423
V_O-Pt/TiO_2	Glass carbon	0.238	~60	33	N.A.	*Angew. Chem. Int. Ed.*, 2020, DOI:10.1002/anie.202104856

Supplementary Table 4. The fitted parameters of the EIS data of the Pt black, Pt/WO_3 and $La_2Sr_2PtO_{7+\delta}$ catalysts for HER.

Catalyst	η (mV)	R_s (Ω)	T (F s^{n-1})	R_1 (Ω)	n_1	R_2 (Ω)	C_φ (F)
Pt black	0	4.65	0.0015	5.7	0.86	3600	0.0036
	-10	4.80	0.0015	4.8	0.85	1650	0.0040
	-20	4.84	0.0022	5.3	0.79	754	0.0055
	-30	4.75	0.0055	4.0	0.68	366	0.0090
	-40	4.55	0.018	4.2	0.46	169	0.022
Pt/WO_3	0	4.78	0.00078	20.2	0.87	3148	0.00077
	-10	5.00	0.00059	20.2	0.90	1470	0.0014
	-20	4.79	0.0016	17.0	0.76	460	0.0030
	-30	4.46	0.0031	12.3	0.62	368	0.0060
	-40	4.71	0.0019	7.1	0.69	92	0.016
$La_2Sr_2PtO_{7+\delta}$	0	4.21	0.00027	574.4	0.85	4454	0.00031
	-10	4.62	0.00030	128.0	0.85	1308	0.0010
	-20	4.56	0.00027	79.4	0.85	665	0.0021
	-30	4.54	0.00024	53.1	0.86	262	0.0043
	-40	4.71	0.00013	59.5	0.91	67	0.013

Supplementary Note 1. Turnover frequency calculations

The active sites per real surface area are calculated from the following formula:

No. of active sites = $(\frac{\text{No.of atoms/unit cell}}{\text{Volume/unit cell}})^{\frac{2}{3}}$

From Figure S8, we can calculate the number of active sites per surface area for Pt and $La_2Sr_2PtO_{7+\delta}$:

No. of active sites (Pt) = $\left(\frac{\frac{4}{\text{unit}}\text{cell}}{60.42\frac{\text{Å}^3}{\text{unit}}\text{cell}}\right)^{\frac{2}{3}}$ = 1.637×10^{15} atoms cm^{-2}

No. of active sites ($La_2Sr_2PtO_{7+\delta}$) = $\left(\frac{\frac{3}{\text{unit}}\text{cell}}{526.01\frac{\text{Å}^3}{\text{unit}}\text{cell}}\right)^{\frac{2}{3}}$ = 3.192×10^{14} atoms cm^{-2}

The real surface area for HER is calculated from the electrochemical active surface area (ECSA), which can be determined by integrating the charge of hydrogen underpotential deposition (H_{UPD}) in cyclic voltammogram (CV) curves according to previous studies.[1, 2] The H_{UPD} integrated areas of $La_2Sr_2PtO_{7+\delta}$and Pt black are 1.99 m^2/g_{Pt} and 70.11 m^2/g_{Pt}, respectively.

Finally, the plot of current density can be converted into a TOF plot according to the following formula:

$$\text{TOF}=\frac{\text{I}}{\text{2Fn}}$$

where I is the current (A) during linear sweep measurement, F is the Faraday constant (C mol^{-1}), n is the number of active sites (mol). The factor 1/2 is based on the consideration that two electrons are required to form one hydrogen molecule.

Supplementary References

1. B. Lim, M. Jiang, P. H. C. Camargo, E. C. Cho, J. Tao, X. Lu, Y. Zhu, Y. Xia, *Science* **2009**, *324*, 1302.
2. M. Li, Z. Zhao, T. Cheng, A. Fortunelli, C. Y. Chen, R. Yu, Q. Zhang, L. Gu, B. V. Merinov, Z. Lin, E. Zhu, T. Yu, Q. Jia, J. Guo, L. Zhang, W. A. Goddard III, Y. Huang, X. Duan, *Science* **2016**, *354*, 1414.
